# Epidemiology and field efficacy of anthelmintic drugs associated with gastrointestinal nematodes of sheep in Nejo district, Oromia, Ethiopia

**DOI:** 10.1038/s41598-024-55611-7

**Published:** 2024-03-21

**Authors:** Latera Solomon, Geremew Haile, Nejash A. Ahmed, Debela Abdeta, Workineh Galalcha, Yacob Hailu

**Affiliations:** 1 Jarso District Agricultural Office, West Wallaga, Nejo, Ethiopia; 2 College of Veterinary Medicine and Agriculture, Addis Ababa University, Bishoftu, Ethiopia; 3 Animal Health Team, Daro Lebu District Agriculture Office, Mechara-Mechata, Ethiopia; 4 Nejo Agricultural Poly Technique College, West Wallaga, Nejo, Ethiopia

**Keywords:** Anthelmintic resistance, Epidemiology, GINs, Nejo, Sheep, Biological techniques, Zoology

## Abstract

Gastrointestinal nematodes (GINs) are major constraints to health and productivity of small ruminants. Methods of their control relies mainly on anthelmintic drugs; however, the indiscriminate use of these drugs could lead to the development of anthelmintic resistance (AR). This study aimed to investigate the epidemiology of GINs infection, and field evaluation of anthelmintic efficacy in sheep. The epidemiological data were collected using a cross-sectional study design while a farm-based field study design was employed for the evaluation of anthelminthic efficacy. Furthermore, standard parasitological techniques were employed for qualitative and quantitative worm identification. The overall prevalence indicated 50.3%. Six genera of GINs (*Haemonchus*, *Trichostrongylus*, *Oesophagostomum/Chabertia, Trichuris*, *Teladosargia/Ostertagia* and *Nematodirus*) were identified. Among the identified genera, *Haemonchus* (25.4%) and *Trichostrongylus* (24.8%) were the dominant genera followed by mixed infection (21.8%), *Oesophagostomum/Chabertia* (10.4%), *Trichuris* (7.8%), *Teladosargia* (*Ostertagia*) (5.7%) and *Nematodirus* (4.1%). Mixed infections consisted either of double infections with *Haemonchus* and *Trichostrongylus*, or triple infections with *Haemonchus, Trichostrongylus* and *Trichuris*. The McMaster egg counting results showed that the mean EPG of infected sheep was 845.6. The results also showed 66 (34.2%), 101 (52.3%) and 26 (13.5%) sheep had low, moderate and heavy worm burden, respectively. Albendazole and Ivermectin showed low efficacy (percentage reductions = 90% and 92%; 95% lower confidence limit = 82.1% and 83.6% respectively) whereas Tetramisole was effective (FECR% = 96.8%; 95% LCL = 93.4%). Factors such as age, body condition, management system and past deworming history of sheep were found to have a statistically significant (p < 0.05) influence on the occurrence and burden of the worms. This is further explained as the highest prevalence and worm burden was detected in sheep of young age (p = 0.008; OR = 0.58; 95% CI = 0.39–0.87), poor body condition (p = 0.001; OR = 0.08; 95% CI = 0.04–0.16) and sheep kept under semi-intensive (p = 0.04; OR = 1.53; 95% CI = 1.02–2.29) with no deworming history for the last two months (p = 0.001; OR = 2.97; 95% CI = 1.94–4.56). The study results revealed that nematode infections were among sheep health constraints that could hurt their productivity while low efficacy of Albendazole and Ivermectin were detected. Therefore, the appropriate management techniques of GIN infections should be designed and implemented. Moreover, a further study involving more sensitive techniques (e.g. Mini-FLOTAC, molecular, and serological techniques) should be conducted by considering different host and environmental risk factors such as production level and seasons.

## Introduction

Ethiopia has about 40 million head of sheep^1^ that are vital to the livelihoods of poor farmers by supplying a variety of products and services like a source of cash income, meat, manure, wool/fiber, gifts, and a variety of other things^2–5^. Because raising sheep requires less input than raising large ruminants, it is thought to be a more reliable source of income for landless and poor farmers^6^. Across the country’s rural communities, selling fattened sheep is a highly profitable and low-risk activity that could employ many jobless young^7^. Despite their contributions, the health and productivity of sheep are constrained due to multiple factors such as drought, the poor genetic potential of the animals, the traditional husbandry, and the prevalence of numerous diseases^8,9^.

Throughout the world, parasitic illnesses continue to be the principal obstacle to animal production, including sheep. Clinical and sub-clinical helminthiasis adversely impair domesticated livestock's productive and reproductive potential. Gastrointestinal nematodes (GINs) have a substantial impact on sheep productivity leading to poor growth, and decreased wool production^10,11^. They cause serious health problems such as anemia, enteritis, gastritis, hemorrhage, diarrhea hypoproteinemia, decreased digestion and absorption, and even death^12–15^. The most economically important genera of GINs in small ruminants are *Haemonchus*, *Trichostrongylus*, *Teladorsagia*/*Ostertagia*, *Oesophagostomum*, *Trichuris*, *Nematodirus* and *Strongyloides*^16–19^.

World-wide, worm control in domestic animals, including sheep, mainly depends on the use of anthelmintic drugs^20^, however, a major constraint on the control of helminth infections in livestock is treatment failure due to anthelmintic resistance (AR)^21–24^. In Ethiopia, the most commonly used anthelmintics for the management of GINs infections in livestock are Albendazole, Tetramisole and Ivermectin. The extensive use of these drugs could lead to the emergence of AR in different nematode populations^25–28^ AR is now widespread in all the major GINs of sheep and goats^29–38^ which is described as a significant threat to their production. AR can occur due to many factors such as under-dosing, frequent and indiscriminate use of drugs and use of anthelmintic with substandard quality compounds^11^. Indiscriminate use of anthelmintic due to paucity of information on epidemiology of worm infections could resulted in the selection of drug-resistant helminth populations. Because there are only a limited number of medications available and discovering new medications is costly, AR presents an alarming global concern that needs to be closely monitored and managed. Even though a few studies have been reported in Western Oromia^39–42^, there is a scarcity of studies that provide information on the epidemiology of GINs and efficacy of commonly used anthelmintic drugs for the control of worm infections in livestock including sheep in the study area. Therefore, the objectives of the present study were (1) to investigate the epidemiology of GINs infections in sheep in Nejo district, West Wallaga zone, Ethiopia. (2) to evaluate the efficacy of commonly used anthelminthic drugs for the control of worm infections in sheep managed under farm condition in Nejo.

## Materials and methods

### Study area description

**Figure 1:**
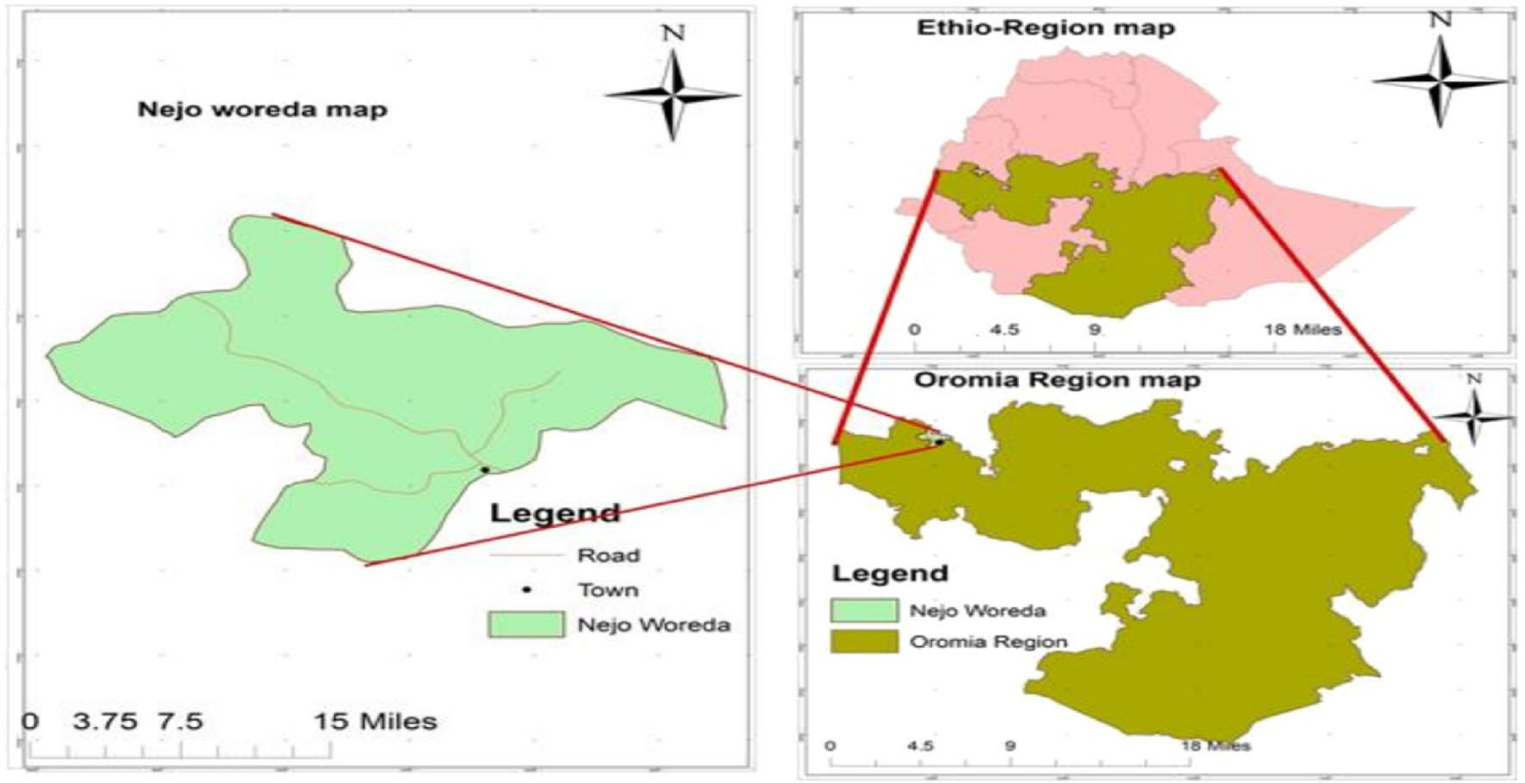
Map of the study area. Source: Hika and Anteneh^44^.

This study was conducted in randomly selected three Peasant Associations in Nejo district (Eba Wakayo, Walitate Agar and Nejo town). Nejo is a district in the West Wallaga Zone of the Oromia Regional State of Ethiopia, which is located approximately 516 km from the capital city, Addis Ababa (Fig. 1). Geographically, the region is situated between 9033′07 N and 35025′50 E latitude and longitude, with an elevation that varies from 1600 to 2200 m above sea level. The area receives approximately an average of 1600–2150 mm of annual rainfall. The mean annual temperature varies from 18 to 28 °C. The livestock populations that are found in the district include 203,028 cattle, 34,477 sheep, 22,733 goats, 504 mules, 25 horses, 9500 donkeys and 101,572 poultry. Livestock in the area is mainly kept under an extensive management system. The mixed crop-livestock farming system was practiced in the area^43^.

### Study design

The study was conducted using a combination of a cross-sectional field study and a farm-based anthelmintic efficacy test between December 2021 to June 2022.

### Study population, sample size and sampling techniques

For epidemiological data, 384 indigenous breed sheep kept under extensive and semi-intensive management systems were included. The sample size was calculated based on the formula given by Thrusfield^45^ with the assumption of 50% prevalence and a 95% confidence interval as there is no prevalence data in the study area. The animals were selected by simple random sampling techniques from each study site. Risk factors such as age, sex, body conditions, management systems, past deworming histories, and study locations were recorded. The age of the sheep was determined based on the dentition given by Solomon and Kassahun^46^ and the owner's information. From day old up to 12 months of age, lambs were considered as young and the rest as adults. Similarly, the body condition of sheep was classified as poor, medium, and good according to the scorecard given by Thomson and Bahhady^47^.

For the anthelmintic efficacy test, a total of 40 sheep from a private farm kept under semi-intensive management conditions were purposively selected after explaining the importance of the study to the owners. The farm comprised 100 local breeds of sheep. The major sources of feed for the sheep were grass and concentrate. Criteria for inclusion were sheep that had not received any anthelmintic in the previous 12 weeks and a fecal egg count (FEC) ≥ 150 eggs per gram of feces based on the World Association for the Advancement of Veterinary Parasitology (WAAVP) guidelines for anthelmintic efficacy and resistance^48,49^. Tetramisole, Ivermectin and Albendazole were commonly used anthelmintics for deworming program.

### Study methodologies

#### Fecal sample collection

About 10 g of fecal samples were collected directly from the rectum of each study sheep into universal fecal bottles. Each bottle containing a fecal sample was labeled with key information such as age, sex, geographical area, and date of sample collection. The fecal samples were transported to Nejo Agricultural Poly Technique College, Animal Health laboratory room using an ice box for laboratory analysis. As fecal culture was used for parasitic identification, any preservative chemicals were not used. The samples were stored at +4 until processed^11,15,50,51^.

#### Fecal flotation technique

Initially, a simple test tube flotation technique using a saturated solution of sodium chloride (NaCl; specific gravity = 1.2) was used for the detection and identification of GINs eggs. Briefly, 3 g of feces of individual sheep was added to a beaker and mixed with 50 ml of flotation fluid using a stirring device. The fecal suspension was filtered through a tea strainer into another beaker. The fecal suspension was poured into test tubes until the meniscus formed and was covered with coverslips. The test tubes were waited for 5 min to allow the eggs to float above the suspension. The coverslips were transferred to the microscopic slides and examined under a light microscope. The eggs were identified based on the morphological characteristics and typical structure of parasite eggs^11,50^.

#### Fecal egg count (EPG)

The modified McMaster egg counting method was used to calculate the EPG of sheep that were confirmed positive for GINs eggs by the flotation technique. Both chambers of the McMaster were filled with the positive sample and examined after 5 minutes. The EPG was calculated by multiplying the total number of nematode eggs counted in each chamber by 50^47^. The infection level was categorized as low (EPG = 50–799), moderate (EPG= 800–1200), and severe (EPG > 1200)^51^.

#### Fecal culture and larval collection

Fecal culture from each individual positive sheep was done to recover third-stage larvae (L3) for further identification of worm genera. Briefly, equal amount of fecal samples were put in a Petri dish and kept at room temperature for 14 days. The samples were mixed regularly to prevent fungal growth. The samples were also checked for moisture and water was added when necessary. After two weeks, the larvae were collected using the Baerman technique by following the procedures described in MAFF^52^.

#### Baerman technique

5 g of the fecal culture was put on a double layer of gauze and thighed. The sample was suspended above the funnel of the Baerman apparatus using a stick and filled with warm water until the fecal material was covered. After 24 h, 15 ml of the suspension was collected into fecal test tubes and centrifuged to decant the supernatant. After removing the supernatant, the drop of sediment was mounted on slides and killed using Lugol’s iodine. Then the slides were covered with coverslips and examined under the microscope at 40×. The parasites were identified to their genera based on morphological characteristics of L3 such as the shape of caudal and cranial extremities, number of intestinal cells, and length of the esophagus and cranial refractile as described by^50,53–55^.

#### Anthelmintic efficacy test

The anthelmintics tested for efficacy were Albendazole bolus 300 mg, 7.5 mg/kg manufactured by Habei Lihua Pharmaceutical Co., Ltd, China; Tetramisole HCL bolus 600 mg, 15 mg/kg manufactured by DIPS, BIOSCIENCE in India and Ivermectin 1% injection, 0.5 ml/25 kg manufactured by Habei Lihua Pharmaceutical Co., Lt, China. The anthelmintics were chosen based on the information that they were the most frequently used anthelmintics in the study area. Forty sheep with no history of treatment with any anthelmintics and EPG > 150 were randomly grouped into four groups (Group-A, I, T, C), each containing 10 sheep. A unique identification code was given to each animal. The weight was individually estimated by using the heart girth method and animals were treated according to their body weight with the dose recommended by the manufacturer. Group-A animals were treated with Albendazole, Group-I with Ivermectin, Group-T with Tetramisole and Group-C was left untreated (control). Albendazole and Tetramisole were administered orally using a small ruminant bolus gun to avoid incorrect administration, whereas Ivermectin injection was given subcutaneously. The first fecal samples were collected from the rectum of each animal on day zero immediately before dosing, and the second rectal samples were taken on day 14 post-treatment for the Percentage in Fecal Egg Count Reduction (FECR%)^35,37,38^. A fecal egg count was performed using the modified McMaster technique. To assess the level of efficacy of the three chosen medications, the FECR% for each group was computed using the following formula^47^.

$${\text{FECR}}\% = \left[ {{\text{Xt}}1 - {\text{Xt}}2} \right]{\text{/Xt}}1 \times 100;$$

where Xt1 and Xt2 are the arithmetic mean of pre-treatment and post-treatment EPG respectively.

Pre-treatment and post-treatment larval cultures were performed on pooled fecal samples for further identification of the nematode genera based on the morphology of the third-stage larvae (L3).

### Data analysis

Data was analyzed using StataCorp. 2015 STATA Statistical Software: Release 14. College Station, TX: StataCorp LPA. Logistic regression and chi-square test models were used to assess the association between the occurrence of GINs and risk factors. The odds ratio and p-values were used to determine the strength and significance of the association. The association between the occurrence of GINs and study variables was said to be significant when p < 0.05 at 95% CI. The efficacy of anthelmintics was evaluated based on the reduction in FECR%. The arithmetic means of pre-and post-treatment fecal nematode egg outputs and 95% upper and lower confidence limits for the reduction were calculated using the approach outlined by Geurden et al.^47^. Low efficacy of Anthelmintic drug is declared only when the FECR% is less than 95% and the lower 95% CL is less than 90%.

### Ethical approval

Ethical clearance was obtained from the research ethics committee of Wallaga University School of Veterinary Medicine on 20 November 2021 (reference number Vet. Med 647/2021). The purpose of the study was explained to the sheep owners, and data were collected after getting permission. Animal experimentation was approved by the research ethics committee and humane animal handling during experimentation and sample collection was applied. Furthermore, all methods were performed in accordance with the relevant guidelines and regulations.

## Results

### Prevalence and risk factors associated with GINs and the effects of risk factors

Of the 384 sheep examined, 193 sheep were positive for one or more groups of GINs with an overall prevalence of 50.3%. The logistic regression analysis showed that study variables such as age, body condition, management system and medication history of sheep had a significant effect on the occurrence of the worm (Table 1). This is explained as young sheep were more affected than adults (p = 0.008; OR = 0.58; 95% CI = 0.39–0.87), sheep with poor body condition were more affected than other body condition score (p = 0.001; OR = 0.08; 95% CI = 0.04–0.16), sheep kept under semi-intensive were more affected (p = 0.04; OR = 1.53; 95% CI = 1.02–2.29) than those kept under an extensive management system. Similarly, sheep with no deworming history for the last two months were more affected than those dewormed (p = 0.001; OR = 2.97; 95% CI = 1.94–4.56). However, statistically significant differences were not observed among the study sites and sex groups (p > 0.05).

**Table 1:** Logistic regression analysis of the association between GINs and respective risk factors.

Risk factors	Category	No. of sheep examined	No. of positive	Percent	Odds ratio	P-value	95% CI
Study site	W/A	137	64	46.7	Ref*		
	E/W	116	65	56.0	1.04	0.726	0.82–1.33
	N/T	131	64	48.9	1.39	0.260	0.90–2.16
Age	Young	177	102	57.6	0.58	0.008	0.39–0.87
	Adult	207	91	44.0	Ref*		
Sex	Male	175	90	51.4	1.00	0.675	0.61–1.37
	Female	209	103	49.3	Ref*		
BCS	Poor	135	109	80.7	1.41	0.001	0.08–0.24
	Medium	172	64	37.2	0.08	0.085	0.04–0.16
	Good	77	20	26.0	Ref*		
M/sys	Extensive	209	95	45.5	Ref*		
	S/intensive	175	98	56.0	1.53	0.040	1.02–2.29
Past D/history	Dewormed	235	94	40.0	Ref*		
	Not dewormed	149	99	66.4	2.97	0.001	1.94–4.56
Total		384	193	50.3			

*W/A* Walitate Agar, *E/W* Eba Wakayo, *N/T* Nejo Town, *BCS* Body Condition Score, *M/sys* Management system, *S/intensive* Semi-intensive, *D/history* deworming history, *CI* Confidence Interval, Ref* reference.

### Identified genera of gastrointestinal nematodes

**Figure 2:**
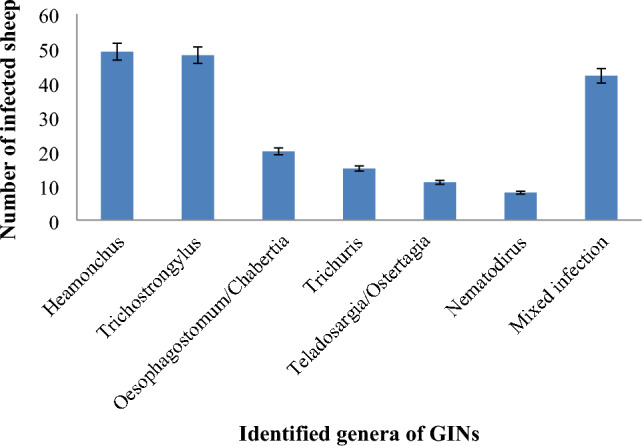
GINs genera identified from fecal culture of sheep.

Six genera of GINs (*Haemonchus*, *Trichostrongylus*, *Oesophagostomum/Chabertia, Trichuris*, *Teladosargia/Ostertagia* and *Nematodirus*) were identified. Amongst the identified genera, *Haemonchus* (25.4%) and *Trichostrongylus* (24.8%) were the dominant genera followed by mixed infection (21.8%), *Oesophagostomum/Chabertia* (10.4%), *Trichuris* (7.8%), *Telodarsagia* (*Oestertagia*) (5.7%) and *Nematodirus* (4.1%) (Fig. 2). Mixed infections consisted either of double infections with *Haemonchus* and *Trichostrongylus*, or triple infections with *Haemonchus, Trichostrongylus* and *Trichuris.*

### Infection intensity

The McMaster egg counting results showed that the mean EPG of infected sheep was 845.6. The results also showed 66 (34.2%), 101 (52.3%) and 26 (13.5%) sheep had low, moderate and heavy worm burden, respectively. The *χ*^2^ test results showed that factors such as the history of anthelmintic deworming, body condition score and management system had a statistically significant relationship with the worm burden (p < 0.05). However, there was no statistically significant difference (p > 0.05) between study sites, age and sex of the sheep (p < 0.05) (Table 2).

**Table 2:** Associations of infection intensity with the respective risk factors.

Risk factors	Category	No. of sheep examined	No. of positive	Level of infection in%			*χ*^2^	P-value
				Light	Moderate	Heavy		
Study site	W/A	137	64	25 (39.0)	28 (43.8)	11 (17.2)	11.6	0.071
	E/W	116	65	21 (32.3)	41 (63.1)	3 (4.6)		
	N/T	131	64	20 (31.3)	32 (50.0)	12 (18.7)		
Age	Young	177	102	36 (35.3)	52 (51.0)	14 (13.7)	7.3	0.063
	Adult	207	91	30 (33.0)	49 (53.8)	12 (13.2)		
Sex	Male	175	90	36 (40.0)	38 (42.2)	16 (17.8)	7.5	0.058
	Female	209	103	30 (29.1)	63 (61.2)	10 (9.7)		
BCS	Poor	135	109	35 (32.1)	57 (52.3)	17 (15.6)	82.4	0.000
	Medium	172	64	21 (32.8)	35 (54.7)	8 (12.5)		
	Good	77	20	10 (50.00)	9 (45.00)	1 (5.00)		
M/sys	Extensive	209	95	32 (33.7)	44 (46.3)	19 (20.00)	11.5	0.009
	S/intensive	175	98	34 (34.7)	57 (58.2)	7 (7.1)		
Past D/history	Treated	235	94	32 (34.0)	50 (53.2)	12 (12.8)	25.6	0.000
	Untreated	149	99	34 (34.3)	51 (51.5)	14 (14.2)		
Total		384	193 (50.3)	66 (34.2)	101 (52.3)	26 (13.5)		

*W/A* Walitate Agar, *E/W* Eba Wakayo, *N/T* Nejo Town, *BCS* Body Condition Score, *M/sys* Management system, *S/intensive* Semi-intensive, *D/history* deworming history.

### Anthelmintic efficacy test

The arithmetic means, Fecal Egg Count Reduction percentage (FECR%) and 95% Upper and lower confidence limit (UCL and LCL) for Albendazole, Tetramisole, and Ivermectin are presented in Table 3. Based on the results, Tetramisole had high efficacy; while low efficacy was recorded for Albendazole and Ivermectin. The fecal culture results showed that *Haemonchus* and *Trichostrongylus* were mostly identified genera followed by *Oesophagostomum*, *Trichostrongylus*, *Trichuris*, *Teladosargia*/*Ostertagia* and *Nematodirus* from sheep treated with Albendazole and Ivermectin (Table 4).

**Table 3:** The efficacy status of Albendazole, Tetramisole and Ivermectin in sheep under farm condition.

Measurements	Treatment groups (number in each group = 10)			
	Control	Albendazole	Tetramisole	Ivermectin
Mean EPG pretreatment	620	628	632	645
Mean EPG post-treatment	570	60	20	50
FERP %	8.8	90.4	96.8	92
95% UCL	–	95	98	96
95% LCL	–	82.1	93.4	83.6
Status of efficacy	–	Low	Effective	Low

*EPG* egg per gram of feces, *UCL* upper confidence limit, *LCL* lower confidence limit.

**Table 4:** The percentages of the worm genera after sheep treated with anthelmintics.

Identified worm genera	Treatment groups (number in each group = 10)			
	Control (%)	Albendazole (%)	Tetramisole (%)	Ivermectin (%)
*Haemonchus*	9 (90)	7 (70)	5 (50)	6 (60)
*Trichostrongylus*	10 (100)	5 (50)	3 (30)	4 (40)
*Teladosargia*/*Ostertagia*	9 (90)	4 (40)	2 (20)	2 (20)
*Trichuris*	3 (30)	3 (30)	2 (20)	3 (30)
*Oesophagostomum/Chabertia*	10 (100)	2 (20)	1 (10)	5 (50)
*Nematodirus*	3 (30)	1 (10)	3 (30)	2 (20)

## Discussion

In the current study, an overall prevalence of 50.3% (193/384) of GIN infections was recorded. This finding was found to be higher than the reports of Abebe et al.^40^ and Aga et al.^56^, who recorded 36.7% and 24.7% prevalence of GINs, respectively. The current findings were nearly similar to the reports of Terfassa et al.^41^ (49.8%) in and around Ambo and Dawit et al*.*^57^ (51.3%) in Hawasa town. However, the finding was lower than the findings of Mulugeta et al.^58^ (91.3%) in and around Bedelle, Sheferaw et al*.*^59^ (83%) prevalence in highland and midland areas of Ethiopia and the prevalence of 88.2% GINs in Córdoba, Colombia by Tachack et al.^60^. The relatively higher prevalence in the current study could be due to a lack of appropriate worm control strategies, development of AR, poor management practices of the animals and frequent exposure to the communal grazing lands that have been contaminated. On the contrary, the relatively lower prevalence compared to the other study sites might be due to the difference in agroecology, diagnostic techniques used and sample size^51,61,62^.

In the current study, six genera of GINs (*Haemonchus*, *Trichostrongylus*, *Oesophagostomum/Chabertia*, *Trichuris*, *Teladosargia* (*Ostertagia*) and *Nematodirus*) and mixed infection were recorded. *Haemonchus* and *Trichostrongylus* are the dominant genera followed by mixed infection, *Oesophagostomum/Chabertia*, *Trichuris*, *Teladosargia* (*Ostertagia*) and *Nematodirus*)*.* Mixed infections consisted either of double infections with *Haemonchus* and *Trichostrongylus,* or triple infections with *Haemonchus, Trichostrongylus* and *Trichuris.* These findings were close to the reports of Bekalu et al.^63^ who reported *Haemonchus* (42.5%), *Trichostrongylus* (38.1%)*, Oesophagostomum* (14.9%), *Bunostomum* (3.7%) and *Trichuris* (0.7%) species in order of their prevalence rate. It is also in agreement with the reports of Sheferaw et al.^59^ and Takele et al.^64^. This may be attributed to the climatic condition of the area, which favors the growth of these parasites, life cycle and species susceptibility. This can be further explained as *Haemonchus* and *Trichostrongylus* species are more common in tropical climates while *Oesophagostomum, Chabertia, Teladosargia*/*Ostertagia* and *Nematodirus* are more common in temperate climates. Moreover, sheep are more susceptible to *Haemonchus* and *Trichostrongylus* than other genera of GINs^11^.

In the current study, the EPG results confirmed that 34.2%, 52.3% and 13.5% of sheep had low, moderate and heavy worm infection, respectively. Lemma and Abera^65^ reported that 30.04% of the sheep were lightly infected, 40.34% moderately infected and 29.62% heavily infected. These results were also consistent with the reports of Abebe et al.^40^ and Terfassa et al.^41^ in Horo Guduru and Ambo, respectively. The immune condition of the sheep, the level of exposure, and the stage of the parasite life cycle could all play a role in the individual differences in parasite burden. In animals with strong immunity female nematodes produce fewer eggs. Animals that come into contact with contaminated grass or water might exposed to many infective larval stages. Moreover, lower fecal egg output might be due to that animals harbor an immature stage of the worm^11,51^. The management system of the sheep had a significant influence on the prevalence of GINs in which higher prevalence was recorded in sheep kept under semi-intensive management than in the extensive management system. These findings agree with the findings of Kusiluka and Kambarage^66^, who reported a low prevalence rate in the open grazing system. This could be attributed to animals being exposed to grass that has been contaminated with worm eggs or larvae from feces^67^.

Even though there was no statistically significant difference, a slightly higher prevalence was recorded in males than females, which was in line with the reports of Dawit et al.^57^. The slight difference between the two sexes might be due to male sheep move a long distance to search for feed which exposes them to infected stages of the worms. In contrast to this, the current findings disagree with the reports of Abebe et al.^40^, Terfassa et al.^41^ and Ousman and Meribo^68^, who recorded higher infection rates in females than in males. This might be because females are more susceptible to parasite infection due to physiological factors such as pregnancy and lactation which lower their immunological responses^11,51,69^.

The current study found that age had a significant influence on the occurrence of GINs in which young sheep were more infected than adults. This agrees with the findings of Abebe et al.^40^; Ousman and Meribo^68^; Dagnachew et al.^70^ and Golo et al.^71^ who reported higher infection in young. The significant differences between the age categories in the current study could be due to lower immune systems in younger than older ones. Animals that are continuously infected with pathogens develop an adequate immune response^65,72–75^. In contrast to this, Tasawar et al.^76^ and Hunde and Chali^42^ reported a higher prevalence of GINs in older animals than in younger ones.

The findings of the current study revealed that statistically significant differences were recorded between the prevalence of GINs and the body condition scores. This is explained as a higher prevalence was observed in sheep with poor body conditions than in sheep with medium and good body condition scores. These findings were in agreement with the reports of Terfassa et al.^41^; Dawit et al.^57^; Hunde and Chali^42^; Belina et al.^74^; Seyoum et al.^77^, who recorded the highest prevalence in sheep with poor body condition scores than other ones. This could be because animals that feed well develop strong immunity against parasite infections. It might be also because worm infections could lead to poor body conditions^78^. However, the presence of significant differences among the three body conditions in this study disagrees with the reports of Nigatu^79^ and Temesgen^14^. In this study, higher worm infection was recorded in sheep not dewormed for the last six months than those dewormed. These findings were in line with the reports of Kenea et al*.*^73^ with a prevalence of 55.4% and 37.0% in untreated and treated sheep, respectively, in Kaffa and Bench Maji Zones, Southwest Ethiopia. It also agrees with the works at Yabello town by Temesgen et al.^14^, who recorded a higher prevalence in untreated sheep than in treated ones. This is because in the study area broad spectrum anthelmintics and ivermectin have been in use for deworming and ectoparasite control.

In the current study, Tetramizole had good efficacy in reducing GINs of sheep, with a FECR% of 96.8%. This finding is in line with the reports of Seyoum et al.^77^; Bahiru et al.^80^; Getachew et al.^37^; Desie et al.^36^; Niguse et al.^81^ and Terefe et al*.*^23^, who reported 97.5−100% efficacy of Tetramizole. However, the studies by Kumar^82^ in Pakistan and Achenef et al.^83^ in North Gondar of Ethiopia found that Tetramizole had the lowest efficacy, with 24% and 74.29% percent reduction, respectively. On the contrary, low efficacy of Ivermectin and Albendazole (FECR% = 90.4, 95% LCL = 83.6) was detected. These findings were comparable to the reports of Sheferaw et al.^84^ in Yabello and Getachew et al.^37^ in the local sheep breed of Areka, Ethiopia, who reported efficacy of 91.8% and 75.8%, respectively, with 95% LCL below 90%. Mondragón-Ancelmo et al.^85^ also reported low efficacy of Ivermectin with 57% FECR% and 95% LCL of 40%. According to Herrera-Manzanilla et al.^86^, the FECR% from 29 to 82% for Ivermectin in Mexico. However, Ivermectin efficacy was reported to be 98.3%, 96.7% and 98%, respectively, by Desie et al.^36^ in Dale district, Seyoum et al.^77^ in the Dabat district, and Hamdullah et al.^87^ in Balochistan which contradicts the current findings.

The suspected low efficacy of Albendazole in the current study was in agreement with reports of Getachew et al.^37^ (57.5% FECR%) in Areka Agriculture Research Centre. Mondragón-Ancelmo et al.^85^ also reported 83% FECR% and 95% LCL of 75% of Albendazole. On the other hand, these findings disagree with the report of Terefe et al.^23^; Seyoum et al.^77^; Bahiru et al.^80^; Achenef et al*.*^83^; Sibhatu et al.^88^ who reported the effectiveness of Albendazole against the treatment of nematode parasites with FECR% of greater than 96%. The low efficacy of Ivermectin and Albendazole in the current study might be due to the strong reliance on the drugs without anthelmintic class rotation, misuse of the drugs and lack of anthelmintic efficacy test. The effectiveness of Tetramisole might be attributed to less frequent use of the drug for deworming in the study area^81,89–93^.

*Haemonchus* and *Trichostrongylus* were mostly identified genera in all treated sheep compared to other genera of GINs suggesting that they are more resistant to anthelmintic drugs. This could be due to the very high prolific potential and high genetic diversity of these genera than other nematodes^11,51^. These findings were in line with the works of Seyoum et al.^77^ and Terefe et al.^23^ who identified more *Haemonchus* and *Trichostrongylus* compared to other GINs after treatment with Albendazole and Ivermectin. *Haemonchus* and *Trichostrongylus* were also found in treated groups with Albendazole and Ivermectin at the study farms in Mexico^86^. According to Achenef et al.^83^. *Haemonchus, Oesophagostomum* and *Trichuris* parasites survive treatment with Albendazole and Ivermectin.

## Conclusion

The finding of this study showed a high prevalence of GIN infections in sheep and this indicates that worm infection is the major constraint to sheep health and production in the areas. This study also identified age, body condition, management system and medication history of sheep had a significant effect on the occurrence of the worm. While the present study indicated Tetramisole was highly efficacious; low efficacy was recorded for Albendazole and Ivermectin. Therefore, the appropriate management techniques of worm infections should be designed and implemented. Moreover, a further study involving more sensitive techniques (e.g. Mini-FLOTAC, molecular, and serological techniques) should be conducted by considering different host and environmental risk factors such as production level and seasons.

## Data Availability

The data that support the findings of this study are available on request from the corresponding author.
